# The Human Retinoblastoma Gene Is Imprinted

**DOI:** 10.1371/journal.pgen.1000790

**Published:** 2009-12-24

**Authors:** Deniz Kanber, Tea Berulava, Ole Ammerpohl, Diana Mitter, Julia Richter, Reiner Siebert, Bernhard Horsthemke, Dietmar Lohmann, Karin Buiting

**Affiliations:** 1 Institut für Humangenetik, Universitätsklinikum Essen, Essen, Germany; 2 Institut für Humangenetik, Christian-Albrechts Universität zu Kiel, Universitätsklinikum Schleswig-Holstein, Campus Kiel, Kiel, Germany; University of Illinois at Urbana-Champaign, United States of America

## Abstract

Genomic imprinting is an epigenetic process leading to parent-of-origin–specific DNA methylation and gene expression. To date, ∼60 imprinted human genes are known. Based on genome-wide methylation analysis of a patient with multiple imprinting defects, we have identified a differentially methylated CpG island in intron 2 of the retinoblastoma (*RB1*) gene on chromosome 13. The CpG island is part of a 5′-truncated, processed pseudogene derived from the *KIAA0649* gene on chromosome 9 and corresponds to two small CpG islands in the open reading frame of the ancestral gene. It is methylated on the maternal chromosome 13 and acts as a weak promoter for an alternative *RB1* transcript on the paternal chromosome 13. In four other *KIAA0649* pseudogene copies, which are located on chromosome 22, the two CpG islands have deteriorated and the CpG dinucleotides are fully methylated. By analysing allelic *RB1* transcript levels in blood cells, as well as in hypermethylated and 5-aza-2′-deoxycytidine–treated lymphoblastoid cells, we have found that differential methylation of the CpG island skews *RB1* gene expression in favor of the maternal allele. Thus, *RB1* is imprinted in the same direction as *CDKN1C*, which operates upstream of *RB1*. The imprinting of two components of the same pathway indicates that there has been strong evolutionary selection for maternal inhibition of cell proliferation.

## Author Summary

Genomic imprinting is an epigenetic process leading to parent-of-origin–specific DNA methylation and gene expression. Defects in this process lead to abnormal development, growth, or behavior. It is still unclear why and how imprinting evolved and how many human genes are imprinted. Based on genome-wide DNA methylation analysis in a patient with a generalized imprinting defect, we have found that the paradigmatic retinoblastoma 1 (*RB1*) gene on chromosome 13 is imprinted. Imprinting of *RB1* is linked to the insertion of a DNA sequence derived by retrotransposition from a gene on chromosome 9. Part of the inserted DNA sequence has evolved into a differentially methylated alternative *RB1* promoter. Differential methylation of this sequence skews expression of the *RB1* gene in favour of the maternal allele. The direction of the imprint imposed on the *RB1* gene is the same as of the maternally expressed *CDKN1C* gene, which operates upstream of *RB1*. The imprinting of two components of the same pathway indicates that there has been strong evolutionary selection for maternal inhibition of cell proliferation.

## Introduction

Genomic imprinting is an epigenetic process leading to parent-of-origin specific DNA methylation and gene expression [1],[2]. Imprints are established during gametogenesis, maintained after fertilization and erased in primordial germ cells (for a recent review see Wood and Oakey, 2006) [3]. It is still a matter of debate, how and why genomic imprinting evolved. The most favoured theory is the kinship theory [4], which postulates a tug-of-war between the two parental genomes over maternal resources during pregnancy and early childhood. As predicted by the kinship theory, several imprinted genes are known to regulate cell proliferation and fetal growth.

To date, ∼60 imprinted human genes are known (http://www.geneimprint.com/). Based on DNA sequence features, Luedi et al. [5] have estimated that there might be some 600 imprinted genes in the mouse. It is likely that a similar number of imprinted genes exist in the human genome. There are at least two reasons why it is difficult to determine the actual number of imprinted genes: (i) imprinted expression can be tissue-specific and (ii) is not always an all-or-nothing phenomenon. The identification of imprinted genes in humans is even more challenging due to experimental limitations. On the other hand, naturally occurring imprinting defects have been identified in human patients, but are unknown in mice. These imprinting defects provide a unique opportunity to identify imprinting control elements, imprinting factors and imprinted genes.

Roughly speaking, imprinting defects are either primary epimutations that occur in the absence of a DNA mutation or secondary epimutations that result from a DNA mutation [6]. Whereas a DNA mutation in an imprinting control region results in a secondary imprinting defect in *cis*, a DNA mutation affecting an imprinting factor typically affects the imprint at several loci in *trans*.

Recently we have observed a patient with hypomethylation of all imprinted loci tested (Caliebe, Siebert et al., in preparation; for clinical details see Materials and Methods). Based on genome-wide methylation analysis of this patient as described here we have found that the *RB1* gene is imprinted. *RB1*, a tumor suppressor gene for the childhood tumor retinoblastoma (accession no. NM_000321) [7], encodes a nuclear phosphoprotein, pRb [8]. When hypo-phosphorylated, pRb acts as a transcriptional cofactor and, by recruiting chromatin remodelling enzymes, represses the proliferation-promoting activities of a subset of E2F transcription factors [7]. Phosphorylation by activated cyclin-dependent kinases (CDKs) results in derepression and activation of E2F dependent promoters. CDK inhibitors such as CDKN1C inhibit this process. In addition to control the G1-S cell cycle transition, pRb has important roles in embryogenesis and maintenance of trophoblast stem cells [9].

Parent-of-origin effects have been reported in human phenotypes associated with mutations of the *RB1* gene. These include differential penetrance and age at onset in retinoblastoma and an excess of first somatic mutations on paternal alleles in sporadic osteosarcoma [10]–[12]. However, as the CpG island/exon 1 region is not known to be imprinted [13], the mechanisms underlying these effects have been unclear.

## Results/Discussion

**Figure 1:**
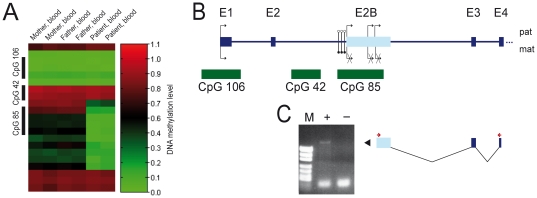
Identification of a novel putative imprinted locus. (A) Heatmap of the Infinium HumanMethylation27 BeadChip (Illumina) for the *RB1* gene. The CpG sites representing CpG 85 show about 50% methylation in DNA from blood of the parents but are hypomethylated in DNA from blood of the patient. In all samples, CpG 42 is methylated and CpG 106 is unmethylated. Target ID of the CpG sites representing CpG 85: cg19427472, cg13431205, cg03085377, cg18481241; CpG 42: cg19447496, cg19296958; CpG 106: cg24937706, cg10552385, cg17055959. (B) Schematic representation of the 5′-region of the *RB1* locus (not drawn to scale) and location of CpG islands (green boxes). Regular exons are shown in blue whereas the new exon 2B is shown in light blue. Open lollipops, unmethylated CpGs; filled lollipops, methylated CpGs; black arrows, transcription start sites. (C) Exon connection PCR. M, DNA length standard; +, with RT; −, without RT; arrowhead indicates the RT–PCR product that was used as template for sequencing; red arrows, location of RT–PCR primers.

In order to identify novel imprinted loci, we performed genome-wide CpG methylation analysis (Infinium HumanMethylation27 BeadChip, Illumina) in DNA from blood of a patient with multiple imprinting defects and appropriate controls. This confirmed hypomethylation of known imprinted loci and, moreover, identified additional loci hypomethylated in the propositus. One of these loci on the array is a 1.2 kb CpG island within intron 2 of the *RB1* gene (CpG 85, UCSC browser, chr13:48,892,636–48,893,857, hg19, http://genome.ucsc.edu; Figure 1A).

**Figure 2:**
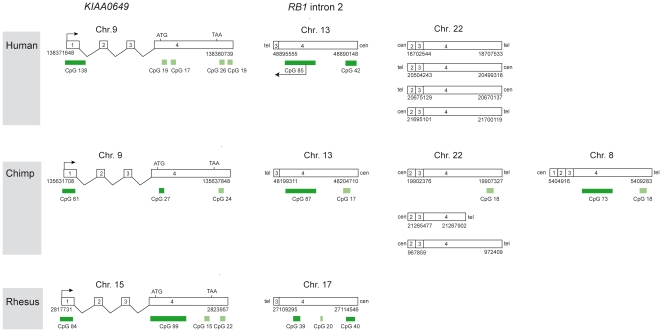
Structure of *KIAA0649* and processed pseudogenes in human (position numbers according to hg19, UCSC), chimpanzee (panTro2, UCSC), and rhesus (rheMac2, UCSC). In the human genome, two of the four small CpG islands in exon 4 of *KIAA0649* (CpG 19 and CpG 17) correspond to CpG 85 in the chromosome 13 copy. The other two (CpG 26 and CpG 19) correspond to CpG 42. The figure also shows the similarities and differences between the situation in humans, chimpanzee and rhesus. Owing to gaps in the chimpanzee and rhesus genome sequences, the picture may not be complete. Light green boxes, CpG islands <300bp; dark green boxes, CpG islands >300bp; arrows, orientation of transcription.

An NCBI Blast search (human build 37 genome data base, http://blast.ncbi.nlm.nih.gov/Blast.cgi) revealed that CpG 85 is part of a 4.5 kb region with a high sequence identity (87%) to exon 4 and 18 bp of the 3′ end of exon 3 of *KIAA0649* (accession no. NM_014811), a four-exon gene in 9q34.3 that encodes a 1209 amino acids protein of unknown function [14]. Four additional intronless copies of *KIAA0649*, each with 89% sequence identity to exons 2 to 4 of the ancestral gene, are located in close proximity to each other on chromosome 22q11.21 (Figure 2). The open reading frame (ORF), which is located in exon 4 of the ancestral gene, is lost in all five processed copies (http://www.ncbi.nlm.nih.gov/gorf/gorf.html). These data suggest that independent retrotransposition events resulted in integration of two processed pseudogenes with different extent of 5′ truncation, one on chromosome 13 and the other on chromosome 22, and that further copies on chromosome 22 are due to gene duplication.

The four small (<300bp) CpG islands present in exon 4 of *KIAA0649* are not present in the pseudogene copies on chromosome 22 but appear to have evolved into two CpG islands (CpG 85 and CpG 42) following integration into the *RB1* gene. Specifically, CpG 85, which spans 1.2 kb, corresponds to the small islands CpG 19 and CpG 17 at the *KIAA0649* locus, which only contain 229 bp and 209 bp, respectively.

By *in silico* analyses (UCSC genome browser and BLAT search) we have found that the processed pseudogene with the CpG island is also present in the *RB1* gene of chimpanzee and rhesus, but not in the *Rb1* gene of mice and rat. As shown in Figure 2, the situation in chimpanzee resembles that in humans with the exception that there is an additional pseudogene copy on chromosome 8, which has a CpG island (CpG 73) of 1.1 kb, and that there are only three pseudogene copies on chromosome 22. In the rhesus the situation is different in that the *KIAA0649* homologue has a CpG island (CpG 99) of 1.5 kb and that there are no other pseudogene copies in the genome apart from the copy within intron 2 of the *RB1* gene, which has a 578 bp CpG island (CpG 39). Based on these data it is possible that the human CpG 85 island has not evolved from two small CpG islands in the ORF of *KIAA0649*, but that a big CpG island in this gene was maintained in the processed pseudogene located within *RB1*, but deteriorated in the ancestral gene as well as in chromosome 22 copies in the human and chimpanzee lineage after the retrotransposition events. Irrespective of whether evolution has shaped or maintained the CpG island within intron 2 of the *RB1* gene, it has acquired a new function (see below).

**Figure 3:**
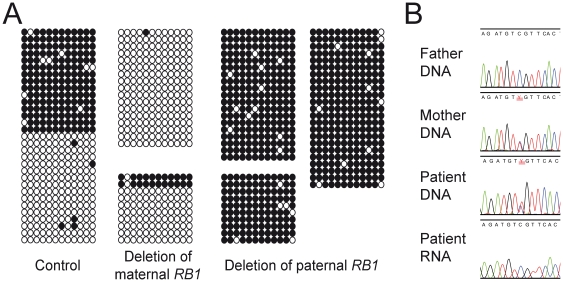
Analysis of CpG 85 and the 2B-transcript. (A) Methylation analysis of CpG 85 by DNA cloning and sequencing. A total of 12 CpG sites within the CpG island were analyzed. Clones from a normal control (blood DNA) were derived from almost fully methylated or unmethylated sequences. Almost all clones from blood DNA from two patients with a deletion of the maternal *RB1* allele were derived from unmethylated sequences, whereas clones obtained from three patients with a paternally derived *RB1* deletion were derived from almost fully methylated sequences. Each block of clones represents an individual. Open circles, unmethylated CpGs; filled circles, methylated CpGs. (B) Allelic expression analysis of the 2B-transcript in blood of a patient heterozygous for a rare variant in exon 3 inherited from the mother. Sequencing of RT–PCR products obtained with primers in exon 2B and exon 3 only showed the paternally derived C allele.

To find out whether CpG 85 is differentially methylated in a parent-of-origin specific manner, we studied 12 CpG dinucleotides after bisulfite treatment, cloning and sequencing of blood DNA from a normal individual and from five retinoblastoma patients with whole *RB1* gene deletions of known parental origin. Because of the high sequence identity between the repetitive sequences, the 248 bp PCR product obtained for subcloning was not specific for the chromosome 13 copy so that sequence differences were used to assign the clones to the different chromosomal regions. By this we found that the chromosome 9 and 22 sequences are fully methylated (data not shown). In contrast, CpG sites in CpG 85 clones from the normal control were either methylated or unmethylated (Figure 3A). Almost all clones from the two patients with a maternal *RB1* gene deletion were derived from unmethylated sequences, whereas the sequence of all clones from the three patients with a paternal deletion indicated fully methylated CpG sites (Figure 3A). We conclude that CpG 85 shows parent-of-origin specific methylation – it is methylated on the maternal chromosome 13 and unmethylated on the paternal chromosome 13. Methylation analysis of two independent sperm samples revealed that the CpG 85 is unmethylated in male germ cells (data not shown).

We also analyzed the CpG island in intron 2 of the chimpanzee *RB1* gene (CpG 87; see Figure 2). Of 29 bisulfite clones sequenced, twelve were derived from methylated sequences, 16 were derived from unmethylated sequences, and one was derived from a partially methylated sequence (data not shown). These findings indicate that the CpG island in intron 2 of the *RB1* gene is differentially methylated in the chimpanzee also.

**Figure 4:**
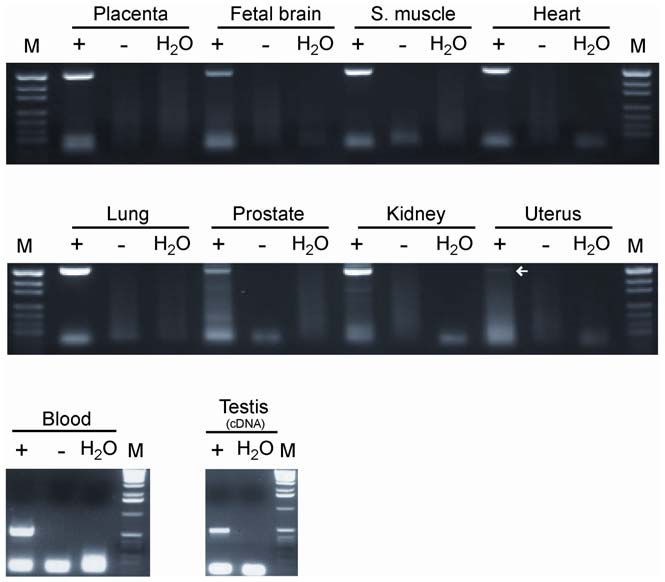
Expression profile of the 2B-transcript. The 2B-transcript is expressed in all of the tissues studied.

To find out if CpG 85 acts as a promoter for an antisense transcript, as is the case for the differentially methylated CpG islands associated with the *Zrsr1(U2af1-rs1)* and *Nnat* genes, for example, which are located in intron 1 of the *Commd1* and *Blcap* genes, respectively [15],[16], we tried to link a spliced antisense EST clone upstream of exon 1 of the *RB1* gene with an unspliced antisense EST clone overlapping with CpG 85. As these experiments as well as 5′- and 3′-RACE (Rapid Amplification of cDNA ends) did not provide any evidence for an antisense transcript, we searched for an alternative sense transcript by exon-connection RT–PCR of CpG 85 and exon 3 and exon 4 of the *RB1* gene. Sequence analysis of the products showed that the CpG island contains a novel start exon (exon 2B) that is spliced onto exon 3 of the *RB1* gene. Three putative transcription start sites were identified by 5′-RACE experiments and, depending on which of them is used, the new exon 2B (Figure 1B) has a size of 478 bp (48893574–48894051), 632 bp (48893420–48894051) or 1159 bp (48892893–48894051). RT–PCR analysis revealed that the 2B-transcript is present at very low levels in many tissues (Figure 4).

We sought to test if parent-of-origin specific methylation of CpG 85 is coupled with monoallelic expression of the 2B-transcript, but as expressed single nucleotide polymorphisms are rare in the *RB1* gene we had to draw on rare variants identified during diagnostic mutation analysis (Table 1). RNA from blood was available from a patient heterozygous for a maternally inherited variant in exon 3 (family A, Figure 3B). Sequence analysis of RT–PCR products specific for the 2B-transcript showed only the C allele, which is of paternal origin.

**Table 1:** Expressed variants in the *RB1* gene.

Fam. ID	Individuals	Location of variants	Genome (L11910)	Protein	Comment	Blood available	LCs available
A	II-1	exon 3	g.39522C>T	p.Ser114Leu	missense	**✓**	**✓**
	III-1					**✓**	**✓**
B	III-1	exon 9	g.61788C>T	p.Thr307Ile	missense	**✓**	**✓**
C	II-1	exon 12	g.70329C>T	p.Asn405Asn	samesense	**✓**	
	III-2					**✓**	
D	II-1	exon 18	g.150009A>G	p.Leu569Leu	samesense	**✓**	
	III-2					**✓**	
E	III-1	exon 21	g.160757T>C	p.Cys712Arg	missense oncogenic	**✓**	
	III-2					**✓**	
F	I-3	exon 21	g.160794T>G	p.Ile724Ser	missense	**✓**	
	II-1					**✓**	
	III-1					**✓**	
G	II-1	exon 23	g.162333C>T	p.Leu819Leu	samesense	**✓**	
	III-2					**✓**	
H	II-1	exon 9	g.61788C>T	p.Thr307Ile	missense		**✓**
	III-1						**✓**

**Figure 5:**
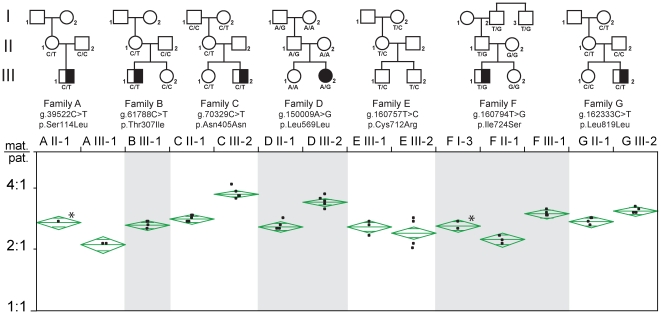
Allelic expression imbalance of the *RB1* gene. Plot of the ratio of allelic expression as determined by SNaPshot primer extension on RT–PCR products obtained from RNA from blood of 14 individuals from 7 families informative for expressed variants (Table 1). The primer extension assay for the variant in exon 3 (family A) only detects the regular transcript whereas the assays for the variants downstream of exon 3 (families B to G) detect transcripts initiated in exon 2B in addition to regular transcripts. Of note, direction and extent of skewing in family A are not different from that in the other families and, therefore, the relative abundance of 2B-transcripts compared to regular transcripts is likely to be low. For each sample 3–5 independent experiments were performed. The top and bottom of the means diamonds represent the 95% confidence intervals for the means. Squares, male individuals; circles, female individuals; filled symbols, bilateral retinoblastoma; half-filled symbols, unilateral retinoblastoma; open symbols, unaffected. Asterisk marks individuals in whom parental origin of alleles is unknown.

**Figure 6:**
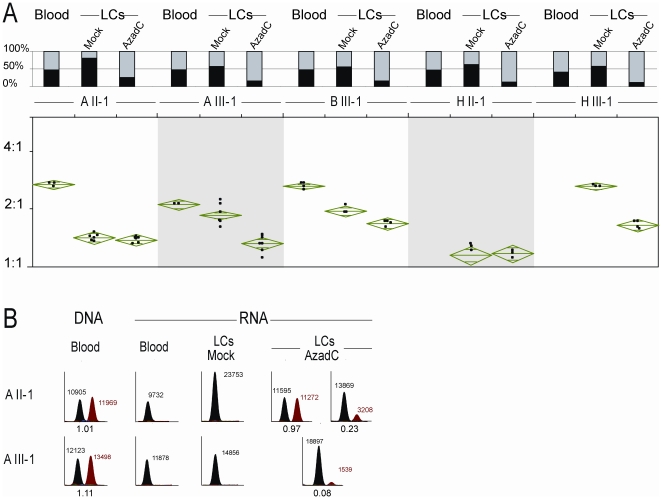
Treatment of lymphoblastoid cells (LCs) with the demethylation drug 5-aza-2′-deoxycytidine (AzadC). (A) Methylation analysis of CpG 85 by methylation-specific PCR (MS-PCR) and quantification of allelic expression imbalance of the *RB1* gene. The top chart shows the methylation status of CpG 85 in blood, mock-treated and AzadC-treated LCs. The percentage of MS-PCR products specific for methylated and unmethylated alleles is indicated by black and grey bars, respectively. The bottom plot shows the ratio of allelic expression as determined by SNaPshot primer extension on RT–PCR products obtained from RNA. For each sample 3–8 independent experiments were performed. The top and bottom of the means diamonds represent the 95% confidence interval for the means. In family H, we could not investigate allelic *RB1* expression in blood, because we did not have RNA from fresh blood. In this family, a male patient with unilateral retinoblastoma (HII-1) inherited the rare variant from his unaffected mother and transmitted it to his unaffected daughter (HIII-1). (B) Electropherograms of SNaPshot primer extension on RT–PCR products specific for the 2B-transcript. Black and red peaks correspond to C and T alleles, respectively. In A III-1, the C allele is known to be of paternal origin. Numbers next to peaks indicate peak areas. Numbers below electropherograms with two peaks show the ratios of peak areas (T-allele/C-allele).

The identification of an alternative *RB1* transcript made from the paternal allele only raised the question whether it is made independently of and in addition to the regular paternal *RB1* transcript. If so, the total level of paternal *RB1* transcripts should be higher compared to that of the maternal transcripts. We investigated this by fluorescence-tagged primer extension analyses of blood RNA from 14 individuals heterozygous for expressed sequence variants of known (n = 12) or likely (n = 2) parental origin (Table 1 and Figure 5). We found allelic expression imbalance in all individuals (2.7±16%, ratio±SD) (Figure 5), but in all cases the imbalance was in favour of the maternal allele. This finding suggested that lack of methylation of CpG 85 and possibly expression of the 2B-transcript interferes with the expression of the regular transcript from the same, i.e. paternal allele. To test this hypothesis, we treated lymphoblastoid cells (LCs) with 5-aza-2′-deoxycytidine (AzadC), which inhibits the DNA methyltransferase DNMT1. Bisulfite sequencing showed that the CpG island associated with the regular *RB1* promoter, CpG 106, was unmethylated in untreated and treated LCs (data not shown). For quantitative analysis of CpG 85 methylation, we established a methylation-specific (MS)-PCR assay. By this we found that in some LCs CpG 85 methylation was greater than 50% (Figure 6A, upper panel). In all cell cultures AzadC treatment had the intended effect of partial loss of methylation at CpG 85.

First, we investigated whether demethylation of CpG 85 resulted in activation of transcription from the maternal 2B-promoter. Analysis of LCs from family A showed that expression of the 2B-transcript remained monoallelic after mock-treatment, as expected (Figure 6B). In two independent experimental rounds, LCs from individuals A II-1 and A III-1 gained biparental expression of the 2B-transcript after demethylation treatment (Figure 6B), although in several assays the expression levels of 2B-transcripts were low or below the detection limit.

Next, we investigated the ratio of the parental *RB1* transcripts. All cell cultures treated with AzadC showed reduced skewing of the allelic *RB1* transcripts (1.4±14%, ratio±SD; Figure 6A, lower panel). The reduction in skewing most likely results from the fact that in many cells the maternal allele has lost methylation and now resembles the paternal allele. Reduced skewing was also observed in the mock-treated LCs (1.7±28%, ratio±SD), which showed some increase in CpG 85 methylation compared to fresh blood. Although there was no strict quantitative correlation between the degree of hypermethylation and the degree of reduction in skewing (Figure 6A, upper panel), the effect was most prominent in LCs from individuals AII-1 and HII-1, which have the highest degree of hypermethylation. In hypermethylated LCs the reduction in skewing most likely results from the fact that in some cells the paternal allele has gained methylation and now resembles the maternal allele. In summary, these results demonstrate that there is a link between allele-specific methylation of CpG 85 and allelic expression imbalance of *RB1*, although they can not provide evidence in favour of a specific mechanism.

We also investigated allelic *Rb1* expression levels in mice, which do not have the intronic CpG island. For this, we crossed FVB and C3H mice, which differ by a single nucleotide (T/C) in exon 25 (rs30444047 at chr.14:73599123), and examined blood from four FVBxC3H and four C3HxFVB offspring by primer extension analysis. The maternal/paternal transcript ratios were 0.69±13% (mean±SD) and 1.49±13%, respectively, which indicate strain-specific effects but no parent-of-origin specific effects on *Rb1* gene expression. This finding strengthens the notion that parent-of-origin specific expression imbalance of the human *RB1* gene is dependent on the presence of the differentially methylated CpG 85.

One possible mechanism for this link is transcriptional interference. As described above, after demethylation of CpG 85 transcription from the maternal 2B-promoter is activated and the *RB1* expression imbalance is reduced. Possibly, the transcription complex binding to the 2B-promoter acts as a road block for the regular transcript [17]. The absence of a quantitative correlation between the amount of the 2B-transcript and the degree of the reduction in skewing may to some extent be due to the fact that 2B-transcript levels are very low and difficult to quantify. However, it may also indicate that the assembly of the transcription complex at the 2B-promoter is more important than the actual transcription.

Another conceivable mechanism is enhancer blocking. Similar to the situation at the *IGF2/H19* locus [18],[19], the unmethylated CpG 85 may bind CTCF or some other insulator protein and block the interaction between the regular *RB1* promoter and a downstream enhancer. So far, however, no such enhancer has been identified, and the *RB1* locus does not contain any experimentally determined *in vivo* CTCF-binding site [20].

In summary, we have shown that parent-of-origin dependent expression imbalance of the *RB1* gene is linked to the insertion of a 5′-truncated, processed pseudogene, which acquired a differentially methylated CpG island. Our findings extend the observations on epigenetically controlled transcriptional interference by retrotransposons [15],[21],[22] to include truncated processed pseudogenes and support the notion that genomic imprinting builds on host defence mechanisms [23]–[26]. A very good example in support of the latter hypothesis is the imprinted *PEG10* gene, which shares homology with an LTR-type retrotransposon, sushi-ichi [27]. Unlike *PEG10*, however, the DNA sequence inserted into the *RB1* gene is derived from an endogenous gene (*KIAA0649*). This appears to be true also for the imprinted *Zrsr1(U2af1-rs1)*, *Nap1l5*, *Inpp5f_v2 and Mcts2* genes, which are located within introns of other genes [15],[22]. These genes are active, independent genes that are likely to encode a protein. In contrast, the *KIAA0649* cDNA fragments must have been dead on arrival, because they lack the 5′-end. They have lost the ORF, and CpG 85 is located within the former ORF. The chromosome 22 copies do not have a CpG island. Thus, the site of integration has determined the evolutionary fate of the cDNA copies.

Of note, the direction of the imprint imposed on the *RB1* gene is the same as of the maternally expressed *CDKN1C* gene, which encodes a cyclin-dependent kinase inhibitor operating upstream of pRb [28]. The imprinting of two components of the same pathway (*CDKN1C* and *RB1*) indicates that there has been evolutionary selection for maternal inhibition of cell proliferation. Neither *CDKN1C* [28] nor *RB1* expression (this work) is strictly monoallelic, probably because complete imprinting would make an individual vulnerable to childhood cancer and would thus have been selected against.

## Materials and Methods

### Ethics statement

The study was approved by the ethics committee of the University Hospital Essen. Blood was obtained after informed consent was given.

### Clinical description

The proband was born after 32 weeks with a weight and length at the 3^rd^ percentile and a head circumference between the 10^th^ and 25^th^ percentiles. After birth umbilical hernia, patent ductus arteriosus requiring surgery, and facial dysmorphism were noted. He has global developmental delay.

### DNA preparation

Human DNA was extracted from blood and lymphoblastoid cells with the FlexiGene DNA Kit (Qiagen, Hilden, Germany) following the manufacturer's instructions. Mouse tail DNA was extracted with the help of EZ1 DNA Tissue Kit for use on the BioRobot EZ1 (Qiagen, Hilden, Germany).

### Bisulfite treatment of genomic DNA

Bisulfite treatment of genomic DNA was performed as described by Kanber et al. [29].

### DNA cloning

PCR products derived from the bisulfite converted DNA were cloned into the pGEM-T easy vector (Promega, Madison, USA). For PCR tagged primers were used: RB1-Ftag; RB1-RM13 (Table S1). PCR conditions were as follows: 95°C for 10 min, 35 cycles of 95°C for 20 sec, 56°C for 20 sec, 72°C for 30 sec, finally 72°C for 7 min. A number of >24 clones were picked and analyzed by DNA sequencing.

### DNA methylation profiling using universal BeadArrays

Bisulfite conversion of the DNA was performed using the “Zymo EZ DNA Methylation Kit” (Zymo Research, Orange, CA) according to the manufacturer's procedure with the modifications described in the “Infinium Assay Methylation Protocol Guide” (Illumina Inc., San Diego, CA). All further analysis steps were performed according to the “Infinium II Assay Lab Setup und Procedures” and the “Infinium Assay Methylation Protocol Guide” (Illumina Inc.). The processed DNA samples were hybridized to the “HumanMethylation27 DNA Analysis BeadChip” (Illumina Inc., San Diego, CA). This array was developed to assay 27,578 CpG sites selected from more than 14,000 genes. Data analysis was performed using BeadStudio software (version 3.1.3.0, Illumina Inc.) using default settings.

### DNA sequencing

Sequence reactions were performed with Big Dye Terminators (BigDye Terminator v1.1 Cycle Sequencing Kit, Applied Biosystems, Foster City, CA, USA) and the cycle sequencing procedure. Reaction products were analyzed with an ABI 3100 automatic capillary Genetic Analyzer and Sequencing Analysis software (Applied Biosystems, Foster City, CA, USA).

### Methylation-specific PCR

The amplification reaction contained 1 µl bisulfite converted DNA in a final volume of 25 µl. Primers used were: RB1-MF, RB1-MR, RB1-UF and RB1-UR (Table S1). Reaction conditions were 95°C for 10 min, 35 cycles of 95°C for 30 sec, 58°C for 30 sec and 72°C for 30 sec, finally 30 min at 72°C. Methylated (maternal) and unmethylated (paternal) product sizes were 126 bp and 119 bp, respectively. PCR products were analyzed on an ABI 3100 Genetic Analyzer.

### RNA preparation

RNA from peripheral human and mouse blood was extracted with either PAXgene blood RNA Kit (PreAnalitiX, Hombrechtikon, Schweiz) or QIAamp RNA Blood Mini Kit (Qiagen, Hilden, Germany). RNA from lymphoblastoid cells was extracted with the RNeasy Mini Kit (Qiagen, Hilden, Germany) following the manufacturer's instructions. To remove residual traces of genomic DNA, the RNA was treated with DNase I (Qiagen, Hilden, Germany).

### Reverse transcriptase PCR

RT–PCRs were performed with the GeneAmp RNA PCR Kit (Applied Biosystems, Foster City, CA, USA). Total RNA from the patients' blood or lymphoblastoid cells was reverse transcribed with random hexamers. For amplification, the Advantage cDNA Polymerase Mix (Clontech, Mountain View, CA, USA) and the GoTaq DNA Polymerase Kit (Promega, Madison, USA) were used. PCR products were checked on an agarose gel and purified either by MultiScreen Filtration (Milllipore, Billerica, MA, USA) or by gel extraction (Wizard SV Gel and PCR Clean-Up System, Promega; QIAquick Gel Extraction Kit, Qiagen). The primers used for the different RT–PCRs are listed in Table S1 and Table S2. For exon connection PCR we designed primers where the forward primer anneals to the CpG island (CpG85-fw) and the reverse primer anneals to exon 4 of the *RB1* gene (RB1-Exon4-rev, Table S1). For amplification we used the Advantage cDNA Polymerase Mix (Clontech, Mountain View, CA, USA). PCR conditions were as follows: 95°C for 1 min, 35 cycles of 95°C for 20 sec, 64°C for 3 min, and finally 3 min at 68°C. For establishing an expression profile of the alternative *RB1* transcript, total RNA from several tissues (Human Total RNA Master Panel II, Clontech, Mountain View, CA, USA) and blood RNA from a normal control was used for the RT–PCR with primers in exon 2B and exon 3 of the *RB1* gene. Expression analysis of human testis was performed on Marathon-Ready cDNA (Clontech, Mountain View, CA, USA).

### Rapid amplification of 5′ cDNA ends (5′RACE)

The 5′RACE was carried out with the 5′/3′ RACE Kit (2^nd^ Generation, Roche, Mannheim, Germany) following the manufacturer's instructions – except of the first-strand cDNA synthesis step. We performed RT–PCR for cDNA synthesis as described above and continued with the next step of the RACE protocol (cDNA purification). Primers used are given in Table S1.

### Cell culture

Lymphoblastoid cells were established by Epstein-Barr virus (EBV) transformation of peripheral blood lymphocytes from the patients and their family members as well as from a normal control. Cells were grown in RPMI 1640 medium with 10% fetal calf serum and 1% penicillin/streptomycin at 37°C.

### Treatment with 5-aza-2′-deoxycytidine (AzadC)

Cells were counted and seeded at an initial concentration of 2.5–3×10^5^ cells/ml in a total volume of 10 ml per flask. The medium was changed every 24 h. A 10 mM stock solution of AzadC (Sigma, St. Louis, MO, USA) was prepared in sterile water and stored in aliquots at −80°C. A daily dose of AzadC (0.5 µM) was added to the flask, whereas control flasks received an identical volume of water. Cells were harvested after 96 h treatment and RNA and DNA were extracted.

### Primer extension analysis

A single nucleotide primer extension method was applied to measure allelic ratios of mRNA (after conversion to cDNA) and genomic DNA (as reference). Using equal amounts of amplicons from cDNA and genomic DNA, quantitative primer extension assay was carried out with ABI Prism SNaPshot ddNTP Primer Extension Kit (Applied Biosystems, Foster City, CA, USA). The SNaPshot reaction products were analyzed by gel capillary electrophoresis on ABI 3700 (Applied Biosystems, Foster City, CA, USA) and electropherograms were analyzed with the Gene Mapper 4.0 software. Allelic DNA ratios were used to normalize the cDNA ratios. Sequences of primers for PCR as well as for SNaPshot are given in Table S2. Means and confidence intervals were calculated with JMP7 (SAS, Cary, NC, USA).

## Supporting Information

Table S1 Primer sequences used for DNA cloning, RT-PCR, methylation-specific PCR, and 5′RACE experiments.
(0.04 MB DOC)

Table S2 Primer sequences for PCR and primer extension analysis.
(0.07 MB DOC)
