# Supplementary material for: The Human Retinoblastoma Gene Is Imprinted
Source: PLoS Genet. 2009 Dec 24;5(12):e1000790. doi: 10.1371/journal.pgen.1000790 (PMC2791201; doi:10.1371/journal.pgen.1000790)
Supplement: Table S1 — Primer sequences used for DNA cloning, RT-PCR, methylation-specific PCR, and 5′RACE experiments. (0.04 MB DOC) [file pgen.1000790.s001.doc]

**Table S1**

| **Method** | **Primer** | **Primer sequence (5’-3’)** | **Annealing temperature** |
| --- | --- | --- | --- |
| Cloning of bisulfite treated DNA | RB1-Ftag | CTTGCTTCCTGGCACGAG-TATATTTGGATGGTTTTTTTAGTGT | 56°C |
|  | RB1-RM13 | CAGGAAACAGCTATGAC-AAACCTCAAATCCAAAATCAC | 56°C |
| Exon connection RT-PCR | CpG85-fw | CTGCCCTTGTTCTCCTGCT | 58°C |
|  | RB1-Exon3-rev | CTGATTTCTATGTTTTTCTGTAGCTC | 58°C |
|  | RB1-Exon4-rev | TCCAATTTGCTGAAGAGTGC | 64°C |
| 5’RACE | 5’RACE-CpG85-2as | CCCATAAAGAGTTCGCCTTTC | 58°C |
|  | 5’RACE-CpG85-3as | CCCAGAAGCCTCAAGTCCAG | 58°C |
| MS-PCR | RB1-MF | GGTTTCGTTTTTTATGGTCGGGTACGGTTTACG | 58°C |
|  | RB1-MR (5’FAM) | AAAAACGTAAAAACGACGACCATACC | 58°C |
|  | RB1-UF | GGTTTTGTTTTTTATGGTTGGGTATGGTTTATG | 58°C |
|  | RB1-UR (5’FAM) | CATAAAAACAACAACCATACC | 58°C |
