# Supplementary material for: The Human Retinoblastoma Gene Is Imprinted
Source: PLoS Genet. 2009 Dec 24;5(12):e1000790. doi: 10.1371/journal.pgen.1000790 (PMC2791201; doi:10.1371/journal.pgen.1000790)
Supplement: Table S2 — Primer sequences for PCR and primer extension analysis. (0.07 MB DOC) [file pgen.1000790.s002.doc]

**Table S2**

| Family ID, exon | Primer | Sequence | Annealing temperature |
| --- | --- | --- | --- |
| Family A, exon 3 | cDNA forward | 5’-AGGACCTGCCTCTCGTCAG-3’ | 55°C |
|  | cDNA reverse | 5’-CTGATTTCTATGTTTTTCTGTAGCTC-3’ |  |
|  | gDNA forward | 5’-TTTTGTTCCCAGGGAGGTTA-3’ | 55°C |
|  | gDNA reverse | 5’-AACGGCTCCATGAGAGAATG-3’ |  |
|  | Snapshot forward | 5’-GCAGCAGTTGACCTAGATGAGATGT-3’ | 54°C |
|  | Snapshot reverse | 5’-TTTTTCTGTAGCTCAGTAAAAGTGAAC-3’ | 54°C |
| Family B,H, exon 9 | cDNA forward | 5’-TTTTATACCTTTTATGAATTCTCTTGG-3’ | 55°C |
|  | cDNA reverse | 5’-GAAGAGTTTTATCATGATCCAAAAATA-3’ |  |
|  | gDNA forward | 5’-TGGGGGATTGACACCTCTAA-3’ | 55°C |
|  | gDNA reverse | 5’-CCTCCCTCCACAGTCTCAAA-3’ |  |
|  | Snapshot forward | 5’-TTTTATGAATTCTCTTGGACTTGTAA-3’ | 51°C |
|  | Snapshot reverse | 5’-CTCTGGAAGTCCATTAGAT-3’ | 41°C |
| Family C, exon 12 | cDNA forward | 5’-TTCCTCCACACACTCCAGTT-3’ | 52°C |
|  | cDNA reverse | 5’-AACCCTGTCCCACAGCTTTA-3’ |  |
|  | gDNA forward | 5’-CCACAGTCTTATTTGAGGGA-3’ | 50°C |
|  | gDNA reverse | 5’-TTCTTTGCCAAGATATTACAA-3’ |  |
|  | Snapshot forward | 5’-AGAAAATCTGATTTCCTATTTTAA-3’ | 52°C |
| Family D, exon 18 | cDNA forward | 5’-TCCCATGGATTCTGAATGTG-3’ | 52°C |
|  | cDNA reverse | 5’-AGTTGGTCCTTCTCGGTCCT-3’ |  |
|  | gDNA forward | 5’-GCCACTGTCAATTGTGCCTA-3’ | 52°C |
|  | gDNA reverse | 5’-AGTTGGTCCTTCTCGGTCCT-3’ |  |
|  | Snapshot reverse | 5’-GATTGTTTAATAAGATCAAA-3’ | 36°C |
| Family E, exon 21,L | cDNA forward | 5’-TTGTGAACGCCTTCTGTCTG-3’ | 52°C |
|  | cDNA reverse | 5’-TGAGGAAGATCCTTGTATGCTG-3’ |  |
|  | gDNA forward | 5’-GAACAAAACCATGTAATAAAATTCTG-3’ | 52°C |
|  | gDNA reverse | 5’-TGAATAAATGAGATCAAATGAATTACC-3’ |  |
|  | Snapshot reverse | 5’-TTAAGGTCTATATTCTTCACTTTGC-3’ | 49°C |
| Family F, exon 21,T | cDNA forward | 5’-TTGTGAACGCCTTCTGTCTG-3’ | 52°C |
|  | cDNA reverse | 5’-TGAGGAAGATCCTTGTATGCTG-3’ |  |
|  | gDNA forward | 5’-GAACAAAACCATGTAATAAAATTCTG-3’ | 52°C |
|  | gDNA reverse | 5’-TGAATAAATGAGATCAAATGAATTACC-3’ |  |
|  | Snapshot forward | 5’-ATAGACCTTAAATTCAAAATCA-3’ | 53°C |
|  | Snapshot reverse | 5’-ATCCTTGTATGCTGTTACA-3’ | 54°C |
| Family G, exon 23 | cDNA forward | 5’-TTCACCCTTACGGATTCCTG-3’ | 52°C |
|  | cDNA reverse | 5’-GGTTTAGGAGGGTTGCTTCC-3’ |  |
|  | gDNA forward | 5’-ATGTAATGGGTCCACCAAAAC-3’ | 55°C |
|  | gDNA reverse | 5’-CAAAATAATCCCCCTCTCAT-3’ |  |
|  | Snapshot reverse | 5’-GTCATTTTTGTTGGTGTTGGCA-3’ | 55°C |
| Mouse SNP rs30444047 | cDNA forward | 5’-TCTCTGCTTTTGCATTCGTG-3’ | 63°C |
|  | cDNA reverse | 5’-TTCACCCTTACGGATTCCTG-3’ |  |
|  | gDNA forward | 5’-CAAGCATCTCCATGGTTCCT-3’ | 55°C |
|  | gDNA reverse | 5’-ATGAAGGCAGAATTGGCATC-3’ |  |
|  | Snapshot forward | 5’-ACCATCTGGTTTATTTTCTGGAACTT-3’ | 56°C |
